# Supplementary material for: Molecular Biomarker Expression in Window of Opportunity Studies for Oestrogen Receptor Positive Breast Cancer—A Systematic Review of the Literature
Source: Cancers (Basel). 2022 Oct 14;14(20):5027. doi: 10.3390/cancers14205027 (PMC9599781; doi:10.3390/cancers14205027)
Supplement: Supplementary file 1 [file cancers-14-05027-s001.zip › cancers-1866621-supplementary.pdf]

Supplementary File S1

1. Breast Neoplasms/
2. Carcinoma, Ductal, Breast
3. Receptors, Estrogen/
4. hormone.mp.
5. ER.mp.
6. Biomarkers/
7. predict*.mp.
8. Pharmacodynamic*.mp.
9. Window*.mp.
10. presurgical.mp.
11. pre-surgical.mp.
12. preoperative.mp.
13. pre-operative.mp.
14. short?term.mp.
15. 1 OR 2
16. 3 OR 4 OR 5
17. 6 OR 7 OR 8
18. 9 OR 10 OR 11 OR 12 OR 13 OR 14
19. 15 AND 16 AND 17 AND 18

Supplementary File S1: Detailed search strategy as used for MEDLINE. Searches on other databases were adjusted in order to fit the specific requirements for each database. Boolean operators "OR" & "AND" were used to combine search terms while asterisks (*) were used to search for variations from a truncated word stem and question marks (?) were used to search for single character substitutions. N.B. Cochrane utilised (*) in place of (?)
